# Management of neonatal complications of macrosomia: A case report at a tertiary hospital in a developing country

**DOI:** 10.1002/ccr3.5298

**Published:** 2022-01-20

**Authors:** Julius Nuwagaba, Darshit Dave

**Affiliations:** ^1^ 281417 Department of Pediatrics Lubaga Hospital Kampala Uganda; ^2^ 242941 Department of Global Health Security Makerere University Infectious Diseases Institute Kampala Uganda

**Keywords:** case report, complications, fetal macrosomia, neonate

## Abstract

Fetal macrosomia can present with numerous complications. We report a case of a term baby girl with a birthweight of 5.31 kg admitted with respiratory distress and suffered several complications of macrosomia. There is a need to closely monitor neonates for early diagnosis and management of complications of macrosomia.

## Abstract 2

Fetal macrosomia is common among neonates and its proper management improves the outcome of these neonates.

## 1 INTRODUCTION

Fetal macrosomia refers to a neonate with a birthweight of ≥4 kg irrespective of gestational age. Other related terms are big baby, and large for gestational age (LGA), known as a birthweight greater than the 90th percentile for gestational age.^1^, ^2^ Macrosomia is associated with several complications: maternal and neonatal complications, occurring either, in utero, during birth, infancy, or even later in life (late complications).^3^ These complications are, however, not only associated with long hospitalization in the neonatal period but also with high morbidity and mortality.^1^, ^4^

The incidence of fetal macrosomia is increasing, and hence, it is apparent that with expounding knowledge and exposure, health workers can take care of a LGA baby and also identify any complications related to a large birthweight. In this article, we present a case of a neonate born at >5 kg who experienced many of the complications of neonatal macrosomia including hypoglycemia, convulsions, respiratory distress syndrome, sepsis, laryngotracheomalacia, and Ebbs palsy due to birth trauma, hypoxic‐ischemic encephalopathy, and polycythemia.

## 2 CASE SUMMARY

A baby girl was born by assisted vaginal delivery to a 40‐year‐old Ugandan multiparous mother at 38 weeks of gestation. The mother spent 9 h in labor. The mother was obese with a body mass index (BMI) of 34.2. The mother had a history of macrosomia during the previous pregnancy; however, she had no history of gestational diabetes and no history of hyperglycemia in this pregnancy. The birthweight of the baby was 5.31 kg, and she had a head circumference of 37.8 cm. The APGAR score at 1 min was 7 and 9 at 5 min. The neonate was admitted to the neonatal unit for monitoring due to macrosomia, mild respiratory distress with tachypnea (76 breaths per minute), and nasal flaring. However, the oxygen saturation was above 94% on admission. The baby was initiated on intravenous (IV) 1 mg of vitamin K stat, IV hydrocortisone at 2 mg/kg thrice daily, and IV 10% dextrose (D10%) at 80 mg/kg/day divided 3 hourly on day one. The baby later in the day developed a fever and had a tachycardia of 154 beats/minute; she was then started on antibiotics: IV amikacin at 15 mg/kg once a day and IV cefotaxime at 50 mg/kg twice a day, as treatment of neonatal sepsis. The baby was also started on 2 hourly breastfeeding as she was tolerating feeds.

After 24 h of hospitalization, the baby developed an episode of generalized tonic‐clonic convulsions. A random blood sugar (RBS) of 1.4 mmol/L was recorded and corrected with bolus 20 ml of IV D10% followed by maintenance IV D10% at 80 ml/kg/day via infusion pump. Bolus IV phenobarbitone was given at a loading dose of 20 mg/kg and maintenance of 5 mg/kg/day. In the due course, the baby had an episode of gasping and arrest of breath, which was restored by 5 rescue breaths, and bag‐mask ventilation, and then put on oxygen therapy via continuous positive airway pressure (CPAP). Gastric aspiration done at this point noted 5 mls of dirty brown aspirates, and the baby was put on Nil per OS (NPO) and initiated on tranexamic acid at 10 mg/kg three times a day. The child was later cycling and fisting, and a cranial ultrasound scan done showed features of mild brain edema. The baby had raised C‐reactive protein on day one, 14.23 mg/L, followed by normal values on subsequent days. She also had deranged serum electrolytes, raised white blood cell count, neutrophilia, and thrombocytopenia on admission (Table 1). However, she had normal renal function tests and liver function tests. An echocardiogram was also done, which showed a patent foramen ovale with a moderate left to right shunting.

**TABLE 1:** Results from laboratory investigations during hospitalization

Parameter	Day 1	Day 2	Day 4	Day 5
C‐reactive protein (mg/l)	14.23		0.8	0.3
Electrolytes (mmol/l)				
Na	130	119	135	
K	5.5	5.6	4.0	
Cl	89	87	95	
Complete blood count				
WBC (×10^3^/µl)	17.72		9.61	
Neutrophils (×10^3^/µl)	11.91		4.26	
Lymphocytes (×10^3^/µl)	3.35		3.76	
Monocytes (×10^3^/µl)	2.16		1.47	
Hemoglobin (g/dl)	20.08		19.83	
Platelets (×10^3^/µl)	44.7		252.6	

As the baby's condition improved, she was found to have a high‐pitched cry and the left arm was medially rotated with the upper arm was extended and pronated. At this point, an impression of macrosomic baby with hypoxic‐ischemic encephalopathy (HIE) stage II complicated with convulsions and respiratory distress, plus neonatal sepsis, thrombocytopenia, and recurrent hypoglycemia. A differential of possible laryngotracheomalacia and Ebbs palsy was also made.

## 3 CASE DISCUSSION

Our patient weighed 5.3 kg at birth, which is >97th percentile of the expected birthweight,^5^ and this alone should prompt a reason for admission to a neonatal unit for early identification of complications and monitoring. Our patient was admitted with mild respiratory distress, but with normal pulse oximetry, a sign of transient tachypnea of the newborn (TTN). Even without complications, macrosomic babies should be admitted for monitoring, especially of their blood sugars as they are at risk of hypoglycemia, a common complication of macrosomia.^6^

Our patient developed hypoglycemia during the course of hospitalization, which triggered her convulsions, one of the most severe complications of low serum blood sugar. Hypoglycemia‐induced convulsions are associated with brain edema, which was also seen in our patient, and this can lead to permanent brain injury especially in cases of recurrent hypoglycemia.^6^, ^7^ It is therefore important that such babies have tight blood sugar control through continuous random blood sugar (RBS) monitoring, administration of 10% dextrose 2 hourly or via infusion pump if available, and topping up with infant formula milk to avoid rebound hypoglycemia in these neonates.^8^

Other complications of macrosomia include birth asphyxia which when severe would lead to hypoxic‐ischemic encephalopathy (HIE),^9^ just like in our patient who had HIE stage II. This was managed by adequate oxygenation and ventilation, key aspects in the management of HIE.

Macrosomia is a major risk factor for neonatal sepsis,^10^ which when coupled with other complications can be a severe form of sepsis, a case in our patient as evidenced by severe thrombocytopenia. Existing antibiotic therapy protocols should guide the choice of antibiotics. Our patient was treated with amikacin and cefotaxime as first‐line and then flucamox and meropenem as second‐line antibiotics based on the guidelines at our hospital's neonatal unit.

An elective cesarean section is the preferred mode of delivery for macrosomic babies. This is a key to preventing complications that can arise from complicated vaginal birth.^11^ The mother, however, insisted on having a vaginal delivery and declined to have a cesarean section. This led to birth compilations, related to trauma during vaginal birth, as the baby was born with a cephalohematoma, injury to the nerve roots (ebbs palsy), and laryngotracheomalacia, all that can be related to complicated vaginal birth. It is therefore important that mothers are counseled on the complications of vaginal delivery for macrosomic babies and elective cesarean sections scheduled especially when the birthweight is >5000 g.^4^

Polycythemia is a complication of macrosomia that was also present in our patient. She had a birth hemoglobin concentration (HB) >20 mg/dl. In some babies, polycythemia can be associated with neonatal jaundice, micro cerebral infarcts, and associated seizures, or even kernicterus and multisystem organ dysfunction.^12^

Other complications of macrosomia to watch out for include metabolic syndrome, meconium aspiration, and meconium aspiration syndrome, skeletal injuries, intrauterine fetal death, childhood obesity, and glucose intolerance with the associated risk of the baby developing diabetes later in life.^2^, ^4^

It is also important to suspect fetal macrosomia by asking for the following risk factors during the antenatal period: maternal diabetes, history of fetal macrosomia, maternal obesity, multiparty, excessive weight gain during pregnancy, maternal age, and postdates (>40 weeks of gestation). The risk factors in our patient were the previous history of fetal macrosomia and maternal obesity.^4^ In case of the aforementioned risk factors, fundal height and obstetric scans can be done to diagnose fetal macrosomia and plan birth accordingly. Pregnant mothers should therefore be active, monitor their weight, and undergo maternal diabetes screening.^13^ In addition, screening strategies for gestational diabetes mellitus (GDM) earlier than 24–28 weeks of gestation should be considered to prevent adverse pregnancy outcomes.^14^

## 4 CONCLUSION

Our report highlights the value of close follow‐up of mothers at risk of fetal macrosomia during antenatal care and counsel them on the ideal mode of delivery, but also close monitoring of the neonates after birth for early diagnosis of complications and their adequate management to reduce morbidity and mortality.

## CONFLICTS OF INTEREST

The authors declare no conflicts of interest.

## AUTHOR CONTRIBUTION

Julius Nuwagaba was involved in the treatment of the patient, patients' follow‐up, conceptualizing the case write‐up, writing the original manuscript, literature review, reviewing the manuscript for important intellectual content, editing supervision, and final approval of the case series. Dave Darshit was involved in writing the original manuscript, literature review, editing, and final approval of the case series.

## ETHICAL APPROVAL

Institutional Review Board approval was not required to publish this article.

## CONSENT

To publish this article, written informed consent was obtained from the parents of the patient.

## Data Availability

Data sharing not applicable – no new data generated.
